# Residents’ Insights on Their Local Food Environment and Dietary Behaviors: A Cross-City Comparison Using Photovoice in Spain

**DOI:** 10.3390/ijerph181910134

**Published:** 2021-09-27

**Authors:** Leyre Gravina, Amets Jauregi, Irrintzi Fernández-Aedo, Julia Díez, Joel Gittelsohn, Uriyoan Colón-Ramos, Manuel Franco

**Affiliations:** 1 Department of Nursing I, Faculty of Medicine and Nursing, University of the Basque Country (UPV/EHU), 48940 Leioa, Spain; amets.jauregi@ehu.eus (A.J.); irrintzi.fernandez@ehu.eus (I.F.-A.); 2 Biocruces Bizkaia Health Research Institute, 48903 Barakaldo, Spain; 3 Public Health and Epidemiology Research Group, School of Medicine, University of Alcalá, 28871 Madrid, Spain; julia.diez@uah.es (J.D.); manuel.franco@uah.es (M.F.); 4 Center for Human Nutrition, Johns Hopkins Bloomberg School of Public Health, Baltimore, MD 21205, USA; jgittel1@jhu.edu; 5 Department of Global Health, Milken Institute School of Public Health, George Washington University, Washington, DC 20052, USA; uriyoan@gwu.edu; 6 Department of Epidemiology, Johns Hopkins Bloomberg School of Public Health, Baltimore, MD 21205, USA

**Keywords:** urban health, food environment, photovoice, participatory action research, inequities, Spain

## Abstract

Perceptions of local food environments and the ability of citizens to engage in participatory research may vary, even if participants share similar cultural and socioeconomic contexts. In this study, we aimed to describe participants’ narratives about their local food environment in two cities in Spain. We used the participatory methodology of Photovoice to engage participants in Madrid (*n* = 24) and Bilbao (*n* = 17) who took and discussed photographs about their local food environment (Madrid; *n* = 163 and Bilbao; *n* = 70). Common themes emerged across both cities (food insecurity, poverty, use of public spaces for eating and social gathering, cultural diversity and overconsumption of unhealthy foods); however, in Bilbao citizens perceived that there was sufficient availability of healthy foods despite that living in impoverished communities. Photovoice was a useful tool to engage participating citizens to improve their local food environments in both cities. This new approach allowed for a photovoice cross-city comparison that could be useful to fully understand the complexity and diversity of residents’ perceptions regardless of their place of residence.

## 1. Introduction

Worldwide, the prevalence of overweight and obesity has increased for the past three decades to an extent that nearly a third of the worlds’ population is obese or overweight nowadays [1]. One of the most important contextual factors associated with obesity is living in poor urban neighborhoods that lack physical and economic access to healthy foods [2]. 

There are many features of the local food environment and related urban conditions that may influence food choices [3]. Unhealthy foods are available in larger portion sizes and at relatively low prices, workers are eating away from home, school food environments provide low-nutrition food [4], and food marketing influences what, where and how much we eat [5,6]. Several studies have linked the food environment with various healthy and unhealthy behaviors [7,8,9] and how those relate to socioeconomic status [10] and educational level [11]. However, individual healthy behaviors may occur only in environments that support these choices by providing access to affordable healthy foods [12,13].

There are underlying factors related to obesity, such as social and political factors [14,15], where we work and the neighborhood where we live [16]. Understanding the determinants affecting obesity development requires studies analyzing how the local food environment relates to residents’ eating habits. Most studies have analyzed this relationship from a quantitative approach [17,18,19,20,21]; however, qualitative studies may provide a deep understanding of where and how people access different food options [22,23]. Qualitative analyses on the influence of the local food environment on food purchasing behaviors [23] have provided a comprehensive understanding of contextual factors’ complexity within community and nutrition environments aiming to further explain consumer dietary behaviors [18,24,25].

Few studies have used a participatory action research approach, such as Photovoice, to stimulate citizens’ critical reflection about community resources and problems by asking them to assess and reflect on their local food environment [26,27,28]. Photovoice is a Participatory Action Research methodology that enables participants to document perceived features of a particular community problem [29]. In Spain, two previous studies have applied the participatory methodology of Photovoice to examine the perceptions of local food environments among residents from low-income areas [8] and socioeconomically diverse neighborhoods [9]. However, so far, no study has assessed two local food environments in different neighborhoods of two different cities in the same country. These studies in Bilbao and Madrid offer now the opportunity to understand if perceptions of local food environments may differ even within the larger similar Spanish culture and social-economic context (middle-low-income areas). To examine this further and more systematically, the current study aimed to compare and contrast perceptions of the local food environment as well as residents’ engagement around these food environments in two different cities of Spain that share a similar cultural and socioeconomic context. This study aimed to answer the following research question: how do perceptions of local food environments compare within shared cultural and socioeconomic contexts in two different cities?

## 2. Materials and Methods

This study emerged from a national collaboration organized by local research teams working in Madrid and Bilbao and community stakeholders in each city, including health technicians, nurses and political decision-makers from public administrations, such as the health department of the regional government and local councils. Both local research teams conducted a Participatory Action Research project using the methodology of Photovoice to understand the key determinants of the local food environments influencing residents’ diets in each city, as detailed elsewhere [8,9]. The current study draws from these efforts and collected narratives and photographs to compare participants’ perceptions of the food environment in each city, how these environments are perceived to be associated with dietary habits and the levels of engagement resulting from the Photovoice project. In brief, the participatory project in Madrid was conducted in 2015 within two neighborhoods: Los Rosales and San Cristobal. In Bilbao, the Photovoice study was carried out in 2017 in the neighborhoods of Uribarri and San Francisco. Both studies were developed in a pre-pandemic context.

Both cities were selected by their similar socioeconomic characteristics and obesity rates of the analyzed neighborhoods. Madrid is the capital city of Spain, with high urbanity and a population of 3.183 million inhabitants. Bilbao is an industrial port city, capital of Vizcaya province, in the autonomous community of the Basque Country, located to the north of Madrid. Bilbao is the largest city in the Basque Country with 345,110 inhabitants. There are unique cultural differences across the region, spanning language, traditions and eating norms, among others [30]. Hence, the inhabitants’ density per hectare of the chosen neighborhoods was similar—403 in Madrid and 427 in Bilbao. These residential areas were selected by level of citizen participation and similar socioeconomic characteristics, lower-middle level. Despite these regional differences, the socioeconomic context of the neighborhoods selected for this study was similar across both sites (e.g., unemployment rate was 16.5 % in Madrid and 13.7% in Bilbao). Moreover, low educational attainment (no primary education) was 34.2% in Madrid and 40.2% in Bilbao. The average income of the neighborhoods in Madrid was 40,195 euros while in Bilbao it was 36,670 euros. Both cities had an ageing population (over 65 years), reflected in 41.8% of Madrid and 40.2% in Bilbao. Adult obesity prevalence in Madrid was 12% similar to Bilbao 13% [31,32,33]. 

Photovoice is a Participatory Action Research (PAR) method whereby participants articulate their needs using photographs to elicit their stories and interpret important issues through discussion groups and critical dialogue [29]. The main goals of Photovoice are: to enable people to record and reflect their community’s strengths and concerns, to promote critical dialogue and knowledge about personal and community issues through large and small group discussions of photographs; and to reach policy makers. Thereby, participatory approaches (like Photovoice) intend to reduce power imbalances between researchers and participants, positioning them as the research topic’s experts [29,34]. Photovoice entails people reflecting on their own community portraits and voices by gathering in five discussion sessions. This technique is a relevant contribution to food environment literature, and even more broadly, to the field of urban health inequalities [35]. Both Photovoice studies used the same purposive sampling strategy and inclusion criteria to engage participants. We invited adults who:

(1) Lived in the neighborhood for more than one year;
(2) Spoke primarily Spanish as primary language;
(3) Had no impediment to manage a digital camera or a smartphone;
(4) Agreed to attend five group discussion sessions.

Our recruitment strategy, in both cities, included distributing information sheets, conducting brief presentations at neighborhood association meetings, or sending e-mails through local neighborhood associations. All eligible residents agreeing to participate completed informed consents, image release forms, and a survey about demographic information. Finally, recruitment resulted in a total of 24 participants in Madrid and 17 in Bilbao. Further details about recruitment strategies can be found elsewhere [8,9].

In brief, four separate photovoice groups, stratified by sex and neighborhood, were set up in Madrid, and two photovoice groups (only stratified by neighborhood) were set up in Bilbao. This was due to the difficulty in recruitment of both genders in Bilbao. Each small discussion group met, at least, for five sessions which were held weekly and lasted approximately two hours. In Section 1, the research team introduced the Photovoice project and encouraged participants “to photograph anything related to their dietary behaviors in their neighborhood”. They also described the schedule of the group sessions and the Photovoice methodology. The ethics-related issues of the Photovoice project (e.g., data protection laws and personal safety) were also discussed and participants were asked to complete a short socio-demographic survey. Finally, they participated in a photography workshop led by a photographer where they were taught how to use their smartphones to take good quality photos. They were instructed to take as many photos as they wanted but asked then to select five pictures to present to the group. During Section 2, Section 3 and Section 4, participants discussed their five photographs and engaged in group discussions. To facilitate these debates, we used the SHOWED questionnaire, which includes five questions: (1) What do you See here? (2) What is really Happening? (3) How does this relate to Our lives/health? (4) Why does this problem or strength Exist? (5) What can we Do about it? [36,37]. After reflecting on the positive and negative aspects of their neighborhood food environment, in session 5, participants agreed upon the final photographs that best defined their local food environment and those to be published in the photo-book. Additionally, they grouped them into topics and more general categories. 

All sessions were audio-recorded (with participants’ permissions) and transcribed verbatim. Two researchers and a health practitioner attended and took notes during the sessions. Following Wang and Burris’ guidelines, data analysis was conducted by the participants themselves in each city [38]. In brief, participants selected the photographs that they perceived as representative of their local food environment; contextualized and critically analyzed the selected photographs guided by the SHOWED questionnaire during the discussion groups; and sorted the photographs into emerging categories based on the discussions. More details can be found elsewhere [8,9].

The results presented in this article derive from participants’ perceptions of their food environment. We collected all transcripts and organized all selected photographs following participants’ categories. Then, we analyzed residents’ narratives using a thematic and in-progress analysis strategy and Atlas.ti. We performed an analytical comparison based on Mill’s qualitative data analysis method, applying the agreement and difference methods, comparing the similar and divergent characteristics between them [39]. Connections were even found between photos of different subjects and they were also taken into account. Afterwards, authors built comparative tables of two or more photos in which similar and different messages and comments were indicated. These were subjected to a peer review [40] whereby another researcher reviewed the work, providing a new analytical point of view to the comparative tables, adding comments and corrections. This way, final comparisons were obtained, as well as the individual photos in which no analogies were found. Finally, the research group met and synthetically extracted similarities and differences between the two cities, choosing a representative photo and narrative by category, in addition to the conclusions.

## 3. Results

Table 1 summarizes the sociodemographic characteristics of participants from both Photovoice projects (*n* = 41).

Participants took a total of 163 photographs in Madrid and 70 photographs in Bilbao. They finally selected 31 photographs in Madrid and 26 photographs in Bilbao. Through their participatory analysis, they identified several emerging thematic categories (34 in Madrid and 18 categories in Bilbao). These were then grouped into five broader themes in Madrid (eating in moderation; cultural diversity; food stores; social relationships; economic crisis and poverty) and into six in Bilbao (unhealthy eating behaviors; cultural diversity; retail transformation; social relationships; precariousness; and healthy eating). Below, we present the themes that were similar and those that were different. As detailed below, we found similar themes in Madrid and Bilbao, with residents in the latter stressing the theme of healthy habits, which was not the case in Madrid.

### 3.1. Similar Themes in Both Cities

#### 3.1.1. Eating in Moderation

**Figure 1:**
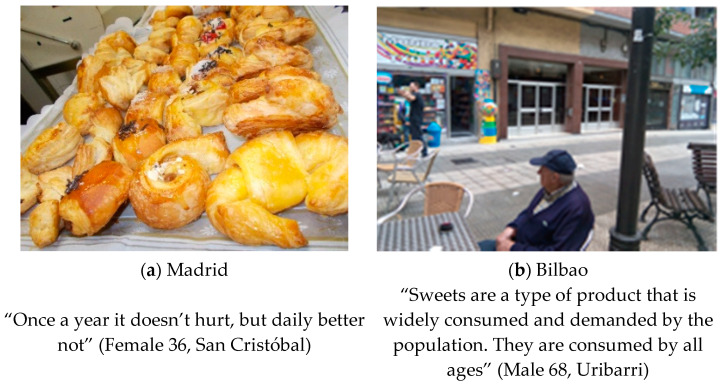
(**a**) Photograph made in Madrid: “At the bakery”. Category: Non processed pastries. Theme: Eating in moderation; (**b**) Photograph made: “Sweet world”. Category: Leisure. Theme: unhealthy eating.

This theme emerged in both cities as their residents commented on the high intake of sugary foods among children and adults (see Figure 1). Beatriz (Female 56, Madrid) when commenting about a candy shop stressed that: “It struck me that there were only adults. It was afternoon and they were women, not children”. Moreover, they agreed that not only sweets, but also fast food was considered as a cheaper and more delicious alternative to healthy food. Rosa (Female 36, Bilbao) explained “There may be a significant abuse of fast food and the fact that more sweets are given to children” and Ana Mari (Female 70, Bilbao) also added, “They are also very capricious; they eat in the street, their parents give them all the whims in the street”.

#### 3.1.2. Cultural Diversity

**Figure 2:**
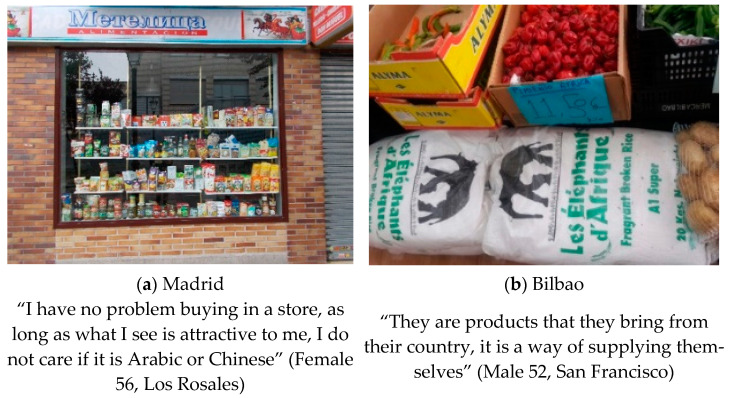
(**a**) Photograph made in Madrid: “Storefront”. Category: food variety. Theme: Cultural Diversity; (**b**) Photograph made in Bilbao: “Elephant products”. Category: Supply. Theme: Multiculturalism.

The second theme that emerged unprompted from the pictures and narratives in both settings was related to the cultural diversity of foods in their neighborhoods. All participants in both settings related cultural diversity to the presence of foreign-born residents in their neighborhoods (see Figure 2). Whilst in Bilbao most residents described how they shopped in foreign resident-owned stores, in Madrid, the theme of cultural diversity, especially regarding shopping in foreign-owned stores, was more varied, with some participants expressing rejection of foreign foods and foreign-owned stores. For example, in Madrid, there were some views of wariness of foreign stores: “I would not enter a Moorish butcher shop, because their culture and hygiene are different from ours”. Sagrario (Female 44, Madrid).

However, there were also perspectives that foreign stores brought in new products that could be tried “what happens, of course, for us is very unknown and if you don’t try it you will never know…”. Beatriz (Female 56, Madrid). See Figure 2a. 

Although in one neighborhood of Madrid (San Cristóbal) the consumption of international products was more normalized, in Bilbao participants described that consumption of foreign foods was for the foreign population mainly. 

“They are products for supplying immigrants, although they are also alternative products for people from here”. However, both agreed that the high availability of international products encourages multiculturalism in the neighborhood. Maribí (Female 41, Bilbao).

#### 3.1.3. Dating during Social Outdoor Outings

**Figure 3:**
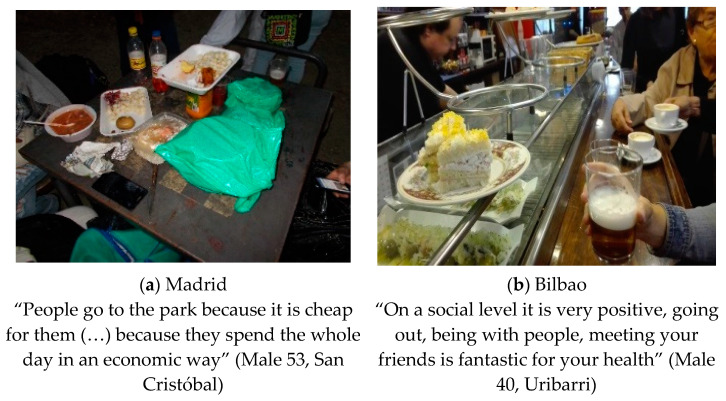
(**a**) Photograph made in Madrid: “Eating in the park”. Category: Street food. Theme: social gatherings; (**b**) Photograph made in Bilbao: “Pintxo-pote”. Category: Pintxos. Theme: Social outdoor outings.

The theme highlighted the importance of eating during social outings at bars, terraces, parks and other outdoor settings, considering that meeting with friends/family outdoors for eating and drinking as being healthy (see Figure 3). Nonetheless, there were differences across settings in how participants described the actual foods consumed during these outings: in Madrid the “pintxos” (portion of food taken as an appetizer, sometimes in skewers) delivered in bars were described as healthy, while in Bilbao participants felt that they were not healthy. As Unai (Male 40, Bilbao) said “From the point of view of nutritional value, it may not be so, since the pintxos are not of a high nutritional quality and are generally accompanied by alcohol”. Additionally, residents from both cities were used to gather in public spaces (like parks) to eat and drink. As Jose Luis (Male 53, Madrid) said “People eating all over the park, because here in San Cristóbal it is common on weekends for people to gather in the park to eat and drink” and Resurrección (Female 71, Bilbao) said “They eat a lot on the street, women and children, all snacks that are made in the store at the time”.

#### 3.1.4. Poverty and Economic Crisis

**Figure 4:**
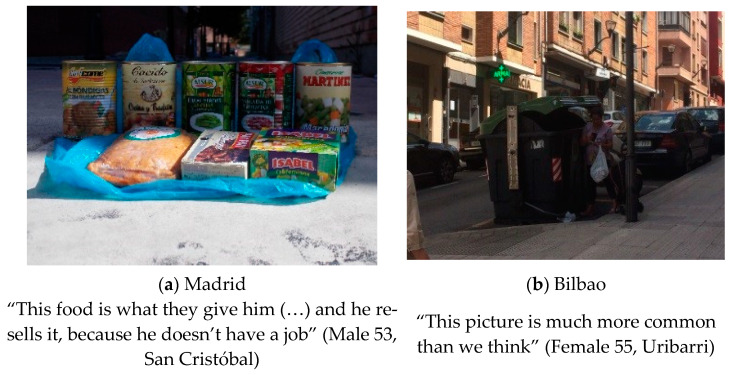
(**a**) Photographs made in Madrid: “Sale of street food”. Category: Illegal street vending. Theme: Economic crisis and food risk; (**b**) Photograph made in Bilbao: “Woman picking up trash”. Category: Insufficient feeding. Theme: Precariousness.

All residents were concerned about the consequences of economic poverty in their neighborhoods and this theme emerged when describing their local food environment (see Figure 4). As Florian (Male 70, Madrid) commented, “what I see is the poverty that exists in the world, in our city (…) you see many people out there rummaging through garbage cans at all hours”. Other residents from both cities described the poverty that prevents a good diet and they also highlighted that price was more important than the quality of the food “It has closed because of the supermarkets that have been built around it. Because of the price, of course, because you buy it somewhere else cheaper, so you stop going there” (Juani, female 46, Madrid). Resurrección (Female 71, Bilbao) also added “The habit of cooking, etc., has to do with your lifestyle. If you do not meet some basic needs, imagine how they will eat”.

#### 3.1.5. Food Retail Perspectives

**Figure 5:**
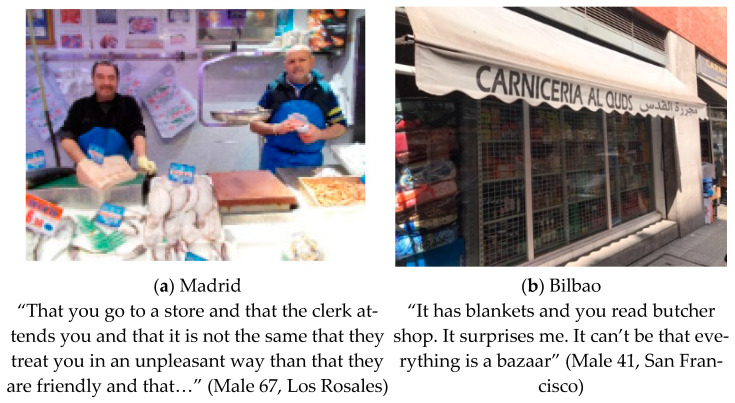
(**a**) Photograph made in Madrid: “The fishermen”. Category: Personal treatment. Theme: The neighborhood store; (**b**) Photograph made in Bilbao: “Butcher with blankets”. Category: Product overcrowding. Theme: Market transformation.

Market transformation was defined by participants in both cities as how any kind of retail store reinvented itself in order to become more profitable. Residents from both cities highlighted their appreciation for the consumption of local products, valuing the small business that offered them confidence, proximity and quality (see Figure 5). As Rosa (Female 51, Madrid) said:

“Closeness and professionalism, and it’s true, I like to go to... I don’t like hypermarkets, big ones, I like small stores” and Alfredo (Male 58, Bilbao) said “It is valuable that these shops have been offering the same products for so many years, which means that they offer quality and offer a guarantee. Also closeness and trust. It is sustainable over time due to these qualities: quality, price, proximity”. 

In other neighborhoods, such as San Cristobal (Madrid) and San Francisco (Bilbao), participants associated multi-product stores with foreign merchants and long hours, coinciding with the unsanitary nature of selling food together with other types of products. 

In terms of bars and restaurants, in San Cristóbal they described few offers and low quality, while in San Francisco there was a great variety and affordability for the level of the neighborhood.

### 3.2. Divergent Themes

#### Healthy Eating Habits

**Figure 6:**
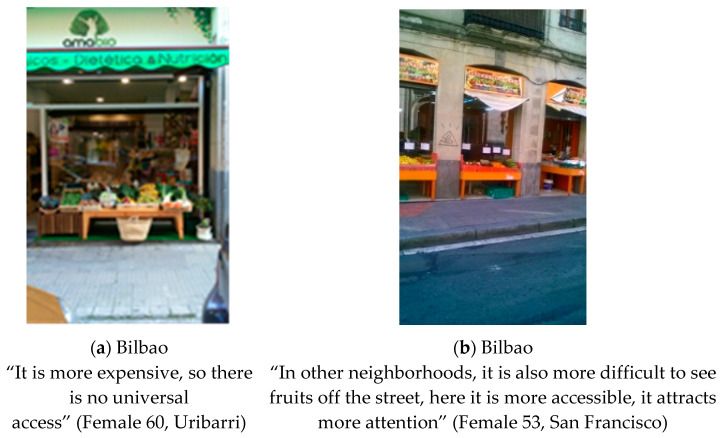
(**a**) Photograph made in Bilbao: “Ecological store”. Category: Original food recovery. Theme: Healthy eating habits; (**b**) Photograph made in Bilbao: “Fresh vegetables”. Category: Restaurants for the neighborhood. Theme: Healthy eating habits.

Although Photovoice groups from both cities mentioned healthy foods, this theme emerged with consensus between the narrative and photographs only in Bilbao, since none of the photos selected by the participants in Madrid related to healthy eating (see Figure 6). After comparing the narratives about this topic, we found that participants related that the organic products were healthier, but less economically affordable. As Mari Luz (Female 60, Bilbao) said “We are raising awareness thanks to food, about the need to eat nearby foods, without chemical and seasonal treatments”. They also talked about how well you can eat even in one of the poorest neighborhoods of Bilbao, as Ana Mari (female 70, Bilbao) told us “You can go there to have a cup of tea, or as it has four little tables at the back, you can eat couscous there or take it home. The food is different but you can eat a lot of vegetables, fruit and fish, with little fat, and they cook beautifully. We generally eat worse, we have eaten fats, pork…”. 

In addition, the food stands exhibiting produce and other foods on the streets of the city were considered by some residents to be a good strategy to promote healthy eating, as Victoria commented (female 53, Bilbao) “In many neighborhoods they don’t let you put the fruit outside, but here it motivates you to buy, the one who is outside with the prices I go looking for the cheapest prices” (see Figure 6b). Although residents from Madrid did not discuss very much about this issue, they did consider negative the food exhibition on the streets due to the poor hygienic conditions. As Daniel (Male 31, Madrid) remarked “This cannot be, having the products on the street where you are going to eat. Apples with this sun that does (…) Where the flies sit, where the dogs pass…”.

**Figure 7:**
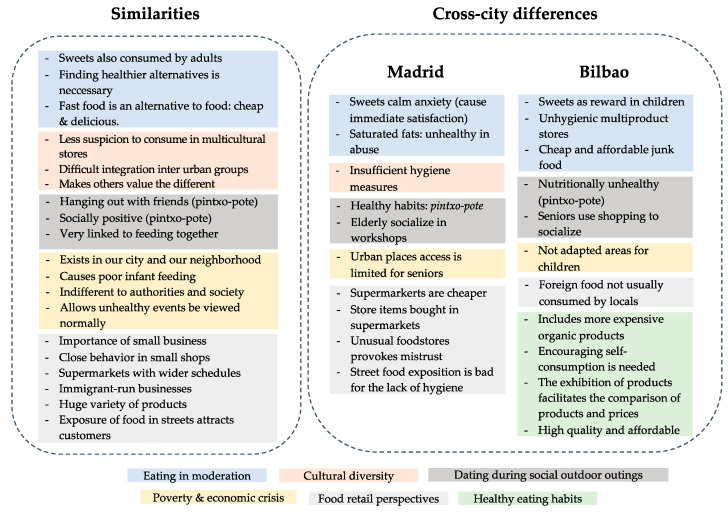
A Cross-city comparison according to the broader themes and the neighborhoods of Madrid and Bilbao made by the authors.

Figure 7 depicts the overall subthemes that, although they emerged in both cities, were interpreted differently. For example, in Madrid, the topic of order to resume the similarities and differences found between both cities, the topic of ‘eating in moderation’ emerged, but in Madrid it was interpreted as sweets calming anxiety and saturated fats as unhealthy if abused, whereas in Bilbao sweets were used as a reward for children, and in addition, they added that unhygienic products and cheap and affordable junk food should be eaten in moderation.

This figure reflects the most recurrent residents’ narratives by emerging themes in each city, Madrid and Bilbao. On one side, the narratives that coincide in both cities are grouped together by colors that indicate each final theme: light blue (eating in moderation), light orange (cultural diversity), dark grey (dating during social outdoor outings), light yellow (poverty and economic crisis), light grey (food retail perspectives) and light green (healthy eating habits).

## 4. Discussion

To the best of our knowledge, this is the first study exploring how perceptions of local food environments did compared within a shared cultural and socioeconomic context using the Photovoice approach. Thereby, we have captured similar but also divergent themes. Residents from both cities reflected on the complex factors influencing their local food environment, such as food insecurity, poverty, cultural diversity, or public spaces used for social gatherings. The differences captured and showed across cities could be influenced by city-level characteristics as well as by the socioeconomic environment. 

Our results highlight that residents from middle-low SES neighborhoods perceived the wide availability of unhealthy foods (e.g., fast food) in their neighborhoods. Thereby, participants from both cities pointed to this vast availability as a facilitator for consumption, in particular, among children. Previous quantitative studies have also shown that access to unhealthy food is higher in low-SES areas as compared to medium-SES and high- SES areas [41,42]. In the city of Madrid, previous studies have also found a higher density of retailers selling unhealthy foods and sugar-sweetened beverages within most socioeconomically disadvantaged areas [43,44]. Moreover, other studies have linked this increased access to a higher consumption of unhealthy food [45,46]. As such, our results depict how the food industry has fabricated food environments where unhealthy products are ubiquitous [47].

In this sense, the association between unhealthy food, attractiveness and palatability was brought to the fore by participants in both cities. Ultra-processed foods were perceived as delicious and as a reward. Previous studies have shown that the food industry uses marketing strategies to encourage this positive image and to lower perceptions of risk [6,48]. Moreover, several harmful substances in food (e.g., added sugar) have been shown to be highly addictive [49]. On the other hand, participants considered healthy foods as unaffordable and thus, less accessible. Indeed, residents valued food affordability over food quality. 

Previous studies have also shown how local food environments are context-specific. In this sense, this study adds new insights to the literature from a resident’s perspective, in terms of context-specific influences. For example, participants from both cities claimed to prefer shopping in specialized retailers (local food stores such as butcheries or fishmongers) than in unspecialized retailers (e.g., supermarkets). They valued the friendly and close way they were treated by sellers and felt confident about buying guarantee and the quality of products. In contrast with these results, several studies have demonstrated that affordable and high-quality foods are more likely to be found in supermarkets than in smaller food retailers [50,51].

Poverty was mentioned as one of the main barriers for having a healthy diet [52]. As shown in the literature, food choice conditions are enabled when enough money, transportation, and retail food outlets are easily available [53]. They showed in photographs the necessity of the poorest ones to rummage through the garbage looking for something to eat or sell. That healthier diets cost more than unhealthy diets is well established in the literature [54,55,56], and so is the argument that food costs influence diet quality that contributes to the appearance of social inequalities in health [57]. Hence, epidemiological studies [58] reveal that obesity occurs and develops mostly in low-income urban and suburban areas [59] and in economically deprived areas [60,61]. 

Precisely, in the low-income areas of our study, the presence of some multi-product stores and bazaars is high and they are considered not hygienic and poor quality. This scenario is worrisome since studies have suggested that proximity to convenience stores and fast-food restaurants has been associated with poor-quality diets [62,63] and with higher body weight [64,65,66]. That is probably why residents could prefer to shop for foods outside their neighborhoods, as it has been shown previously [26]. 

Moreover, the way the foods are displayed to the public would make a difference in residents’ food choices. For example, the exhibition of healthy food in the streets was considered in one city a better opportunity of increasing their healthier intake, while in another city was criticized for lack of hygiene. Hence, not only marketing [67] but also the use of public areas for selling could be a determinant factor of food choices. There is increasing evidence that living and consumption environments are crucial to changing nutritional patterns and maintaining healthier lifestyles [68]. According to the latest national study on eating habits in Spain [69], Bilbao showed better dietary habits than Madrid, eating 11.6% more vegetables, salads and greens every day, and eating 11% more fish, 7.8% more legumes, and 5.5% more fruit or vegetable juices three times or more a week. However, in terms of consumption of unhealthy foods, Bilbao also had a higher daily consumption of sausages and cold meats (5.9%), sweets (17.4%) and fast food (6%). Madrid had a higher consumption of red meat (11.8%), soft drinks with sugar (2.5%), and snacks or savoury finger foods (1.3%) more than three times a week.

Participants also stressed the social and cultural dimensions of dietary behaviors. Eating is perceived as a social activity [70]. Thus, examining it as a social practice gives us an idea of how people connect in the social world and how they generate population eating patterns [53]. In our study, residents remarked on the essential social habit of meeting with family and friends at bar/restaurants/terraces/parks to eat and drink. Going to eat “pintxos” from one bar to another with friends/family was a typical social habit of residents in Spain. Even though “pintxos” were considered expensive and low nutritional quality food, they were consumed frequently and often accompanied by alcoholic beverages, such as beer or wine. These results are related to a previous study showing that social behaviors have a meaningful impact on their food choices [71]. Another way of socializing and knowing other cultures is by shopping in food stores run by foreigners. The remarkable presence of foreign-born residents allows residents to choose among a huge variety of international food products. Although the supply of these products is mainly targeted for specific ethnic groups in the neighborhood, the purchase of exotic products is becoming more normalized in these cities. 

Finally, we want to highlight the strengths of this study. Comparing two photovoice projects across two cities involved the collaboration of two research groups with each other and with the participants. Both groups have received the same training in the use of Photovoice and have implemented the same study protocol. Although citizen participation in each study has been different, the number of participants does not influence participation in this type of participatory study [36]. Besides, the participants’ groups have been heterogeneous considering race/ethnicity, gender, age, educational level, employment, and income. Analyzing the results of each study gives us a better exploration of the local food environment and its influence on the population’s eating habits. All this implies a new area to work in for Public Health services, as it provides a new method of participatory analysis of the inter-city environment based on the investigation of the citizens of their environment. This community action project has promoted the spread of residents’ voices and enhanced critical thinking through the population and policy makers. Public photo exhibitions and citizen gatherings were organized in both cities, and two photobooks were published. All this favors citizen engagement for the development of public policies led by citizens to improve their local urban environment to improve their health.

However, the study has several limitations. First, the study was carried out in two intentionally selected neighborhoods of low-middle socioeconomic level, so the results may not be extrapolated to the entire population of both cities. Secondly, Bilbao did not separate photovoice groups by gender. However, no gender differences were found after analyzing their narratives. Although the sample size may seem small, it is appropriate for Photovoice projects in public health research [36]. However, we would like to highlight that citizen participation varied between cities. Bilbao participants ended up being of slightly higher socioeconomic status. Thus, there was not equal representation of men and women between cities. Third, participants could only select five of their photographs in the small group discussion session, although they could choose which photographs to share and discard. Fourth, we did not include policy-makers from the beginning of the process. By doing so, it could be a mechanism that improves the uptake of the policy recommendations.

## 5. Conclusions

Photovoice proved to be a suitable method for comparing and contrasting residents’ perceptions about their local food environment across different cities within a shared cultural and socioeconomic context. This cross-city comparison highlighted several similar themes influencing residents’ dietary behaviors, such as eating in moderation (especially ultra-processed and sugary foods), cultural diversity, social outdoor outings, poverty and economic crisis and type of food retailers. Our results suggest that participatory methodologies like Photovoice can help designing public health policies and interventions to reverse current obesogenic food environments in Southern European urban contexts.

## Figures and Tables

**Table 1:** Socio-demographic characteristics of the participants (*n* = 41) by neighborhood and city.

	Madrid		Bilbao		Participants (*n* = 41)
	Female *n* = 14	Male *n* = 10	Female *n* = 9	Male *n* = 8	
Foreign-born	3	2	1	3	10
Highest level of education					
College degree	1	1	1	3	6
High-school graduate	5	4	8	3	20
Not a highschool graduate	8	5	1	3	17
Employment					
Employed	1	2	5	6	14
Unemployed	4	4	2	-	10
Retired	3	3	2	2	10
Housewives	6	-	1	-	7
Median household income/month					
<600 €	2	2	3	1	8
601–1200 €	5	4	2	1	12
>1200 €	7	4	4	6	21
Living with spouse or partner in their household	11	9	3	4	27
Primary food purchaser for their household	12	6	6	4	28

Source: The authors.

## Data Availability

The data presented in this study are available upon request from the corresponding author. The data are not publicly available for privacy and ethical reasons. The data were stored in compliance with the principles set out in the Declaration of Helsinki and with Spanish legislation on personal data protection. Other publicly available data presented in this paper was also used provided by the Bilbao Council, the Eustat-Basque Statistics Institute and the Madrid Municipal Register Statatistics.
